# Influence of Environmental Factors and Genome Diversity on Cumulative COVID-19 Cases in the Highland Region of China: Comparative Correlational Study

**DOI:** 10.2196/43585

**Published:** 2024-03-25

**Authors:** Zhuoga Deji, Yuantao Tong, Honglian Huang, Zeyu Zhang, Meng Fang, M James C Crabbe, Xiaoyan Zhang, Ying Wang

**Affiliations:** 1 Research Center for Translational Medicine Shanghai East Hospital School of Life Sciences and Technology, Tongji University Shanghai China; 2 Information School The University of Sheffield Sheffield United Kingdom; 3 Department of Clinical Laboratory Medicine Center Yueyang Hospital of Integrated Traditional Chinese and Western Medicine, Shanghai University of Traditional Chinese Medicine Shanghai China; 4 Department of Laboratory Medicine Shanghai Eastern Hepatobiliary Surgery Hospital Shanghai China; 5 Wolfson College Oxford University Oxford United Kingdom; 6 Institute of Biomedical and Environmental Science & Technology University of Bedfordshire Bedfordshire United Kingdom; 7 School of Life Sciences Shanxi University Shanxi China

**Keywords:** COVID-19, environmental factors, altitude, population density, virus mutation

## Abstract

**Background:**

The novel coronavirus SARS-CoV-2 caused the global COVID-19 pandemic. Emerging reports support lower mortality and reduced case numbers in highland areas; however, comparative studies on the cumulative impact of environmental factors and viral genetic diversity on COVID-19 infection rates have not been performed to date.

**Objective:**

The aims of this study were to determine the difference in COVID-19 infection rates between high and low altitudes, and to explore whether the difference in the pandemic trend in the high-altitude region of China compared to that of the lowlands is influenced by environmental factors, population density, and biological mechanisms.

**Methods:**

We examined the correlation between population density and COVID-19 cases through linear regression. A zero-shot model was applied to identify possible factors correlated to COVID-19 infection. We further analyzed the correlation of meteorological and air quality factors with infection cases using the Spearman correlation coefficient. Mixed-effects multiple linear regression was applied to evaluate the associations between selected factors and COVID-19 cases adjusting for covariates. Lastly, the relationship between environmental factors and mutation frequency was evaluated using the same correlation techniques mentioned above.

**Results:**

Among the 24,826 confirmed COVID-19 cases reported from 40 cities in China from January 23, 2020, to July 7, 2022, 98.4% (n=24,430) were found in the lowlands. Population density was positively correlated with COVID-19 cases in all regions (ρ=0.641, *P*=.003). In high-altitude areas, the number of COVID-19 cases was negatively associated with temperature, sunlight hours, and UV index (*P*=.003, *P*=.001, and *P*=.009, respectively) and was positively associated with wind speed (ρ=0.388, *P*<.001), whereas no correlation was found between meteorological factors and COVID-19 cases in the lowlands. After controlling for covariates, the mixed-effects model also showed positive associations of fine particulate matter (PM2.5) and carbon monoxide (CO) with COVID-19 cases (*P*=.002 and *P*<.001, respectively). Sequence variant analysis showed lower genetic diversity among nucleotides for each SARS-CoV-2 genome (*P*<.001) and three open reading frames (*P*<.001) in high altitudes compared to 300 sequences analyzed from low altitudes. Moreover, the frequencies of 44 nonsynonymous mutations and 32 synonymous mutations were significantly different between the high- and low-altitude groups (*P*<.001, mutation frequency>0.1). Key nonsynonymous mutations showed positive correlations with altitude, wind speed, and air pressure and showed negative correlations with temperature, UV index, and sunlight hours.

**Conclusions:**

By comparison with the lowlands, the number of confirmed COVID-19 cases was substantially lower in high-altitude regions of China, and the population density, temperature, sunlight hours, UV index, wind speed, PM2.5, and CO influenced the cumulative pandemic trend in the highlands. The identified influence of environmental factors on SARS-CoV-2 sequence variants adds knowledge of the impact of altitude on COVID-19 infection, offering novel suggestions for preventive intervention.

## Introduction

### Background

In recent years, the outbreak of COVID-19 has had substantial impacts on human health, social life, and economic trends worldwide. To comprehensively explore the impact of potential factors on the strength and speed of viral infection, we first used the zero-shot model to screen the literature related to broad respiratory infectious diseases, including COVID-19, to identify the major influencing factors through text mining [1]. A significant ranking list of environmental factors was obtained (Multimedia Appendix 1), in which temperature and atmospheric pressure were highly correlated with respiratory viruses. Earlier findings showed that the spread of COVID-19 was linked to various factors, including the environment, sequence variants of the virus, and government countermeasures to protect public health in the face of outbreaks [2,3].

Previous studies also investigated the correlation between COVID-19 and altitude. COVID-19 transmission in high-altitude regions appears to differ from the global pattern, with a lower number of cases reported at high altitudes [4-9]. Moreover, population density was identified as a basic factor that significantly catalyzed the spread of COVID-19 in numerous countries, including India, the United States, China, and Malaysia [10-13]. However, few studies have performed an in-depth analysis of the effects of population density, altitude, and environmental factors on the variation in the severity of the COVID-19 pandemic among regions at different altitudes. 

Moreover, virus mutation is another important factor in escaping the immune protection derived from a previous infection or vaccination [14-16]. Several studies have shown that people living at high altitudes may be less susceptible to developing severe adverse effects from COVID-19, along with reduced case fatality rates [4,8]. Environmental factors have also been shown to actively influence virus mutation and to play regulatory roles in viral evolution [17-20]. Nevertheless, the underlying physiological mechanism linking virus mutation and altitude that could affect the rate of COVID-19 transmission remains unclear.

### Purpose

To fill this research gap, we aimed to explore the potential factors contributing to the outcome of COVID-19 at high altitudes in China in comparison to the lowlands. Toward this end, we first assessed the contributions of altitude and population density on the total number of COVID-19 cases at different altitudes, and further explored the correlation between COVID-19 cases and environmental factors.

In addition, a more detailed correlation analysis among regions at different altitudes was performed at the city level using mixed-effects multiple linear regression models controlling for potential covariates, including meteorological and air quality factors. Furthermore, we studied the genome diversity, mutation frequency, and correlations with environmental factors in high- and low-altitude regions. Overall, we aim to provide a better understanding of the key factors that could influence the cumulative infection and transmission rate of COVID-19 in high-altitude regions of China, which can in turn help to inform establishing improved policies for preventive interventions.

## Methods

### Study Area

We focused on the high-altitude southwestern regions of China and several low-altitude regions from mainland China. A more detailed description of each province included in the study area along with city-level information is provided in Multimedia Appendix 2.

### Collection of Confirmed COVID-19 Cases

Data on COVID-19, including total confirmed cases at the province level from January 23, 2020, to July 7, 2022, were collected from the Dingxiangyuan (DXY-DX Doctor) website [21]; historical cases at the city level over the same study period were collected using the R package “nCovid2019.” The population density for each region was calculated by the following equation:

Population density = population size/area of the land (km^2^) **(1)**

The normalized daily number of confirmed COVID-19 cases was calculated using the following equation:

Normalized COVID-19 cases = total number of confirmed cases/population density **(2)**

To reflect the COVID-19 infection situation in high-altitude regions of China during the study period, descriptive statistics were compiled for the daily average meteorological and air quality parameters, as shown in Multimedia Appendix 3.

### Collection of Population, Meteorological, and Air Quality Factors

To analyze the correlation between altitude and COVID-19 cases in China, altitude information of highland regions (>1500 m) and lowland regions (<1500 m) was collected from the topographic map [22]. Meteorological factors were collected from the World Weather Online site [23]. Air quality variables were collected from the Statistical Yearbook for each municipality or province, along with associated data on population and land areas. Multimedia Appendix 4 provides a more detailed description of all these variables.

### Collection of SARS-CoV-2 Genome Data

SARS-CoV-2 whole-genome sequencing data were collected from the GISAID (Global Initiative on Sharing All Influenza Data) website [24], including 300 sequences from high-altitude regions and 300 sequences randomly selected over the same time period from low-altitude regions. The accession numbers of the sequences included in this analysis are listed in Multimedia Appendix 5.

### Statistical Analysis

The Kolmogorow-Smirnov test was performed for each variable (see Multimedia Appendix 6) with the null hypothesis of a normal distribution; since the *P* value of each variable was less than .05, the null hypothesis was rejected, indicating that the test distribution was not normally distributed. Therefore, we used the nonparametric Spearman correlation coefficient for the correlation analysis of environmental factors and mutation frequency with COVID-19 cases using SPSS software.

After taking into account the correlations for each of the independent variables, a mixed-effects multiple linear regression was used for an adjusted correlation analysis, with the model defined as follows:

Y = Xβ + Zµ + ∈ **(3)**

where Xβ represents the fixed-effects set in this study, including the covariates meteorological and air quality, and Zµ represents an N×M design matrix containing each individual group (N) for each covariate (M) of the random effects. Four sets of mixed-effects models were analyzed using R software (version 4.2.1). Separate models were first run for each of the 12 random effects (environmental covariates), followed by two separate models that included all 6 meteorological indicators or air quality indicators as fixed effects simultaneously. The final model included all 12 indicators simultaneously.

### Genome Analysis

The nucleotide sequences of the whole genome of SARS-CoV-2 were aligned to the reference sequence Wuhan-1 (NC_045512.2) using minimap2 2.17-r974. All mapped sequences were merged back with all others in a single alignment bam file. Variant calling was performed using bcftools mpileup v1.91. Gene sequences of SARS-CoV-2 were extracted and translated into amino acid sequences, which were aligned to the reference sequence by ClustalW. Variant calling was computed using an in-house–developed R script.

### Sequence Diversity Calculation

Sequence diversity was calculated using the Shannon entropy (Sn) index in R software (version 4.2.1), which measures the diversity of nucleotides, amino acids, and their respective variant frequencies. The diversity of each nucleotide position (nucleotide 1 to 29,903) was calculated as the Sn according to the following formula [25]:

Sn = i∑(pilnpi)/lnN **(4)**

where pi represents the relative frequency of nucleotides or deletion at this position and N represents the total number of sequences. All high-altitude samples were compared with the low-altitude samples to identify differential sequence variations at both the nucleotide and amino acid levels.

### Ethical Considerations

This study is based on an analysis of existing data and therefore received exemption from ethical approval (reference number 041797). Some data sets were slightly modified after the study received exemption from ethical approval; however, the updated data were collected from the same publicly available database. According to the University Research Ethics Policy, “the project does not involve human participants which will only use publicly available anonymized data; a project which will only use existing clinical or research data that has been robustly anonymized such that it no longer constitutes personal data” [26] (pp. 3.1.10, p. 20-21); therefore, ethical approval was not required for the above circumstance. In addition, Article 32 of the Ethical Review Measures for Human Life Science and Medical Research of the People’s Republic of China declares that “research involving human life science and medicine under the circumstances of using anonymized information data, legally obtained publicly available data, or data generated through observation without interference with public behavior, which do not cause harm to the human body and do not involve sensitive personal information or commercial interests, can be exempt from ethical review to reduce unnecessary burdens on researchers and promote the development of human life science and medical research” [27] (chapter 3). Therefore, this study falls within the exempt category from the institutional review board. Further ethical approval by a review committee was not required since the data utilized in this study have been sourced from a publicly accessible database, ensuring full anonymization, and the research process involved no direct interaction with human subjects, solely relying on the analysis of pre-existing and publicly available data, as mentioned in the above legislation.

## Results

### Correlation Between Altitude and COVID-19 Cases

**Figure 1:**
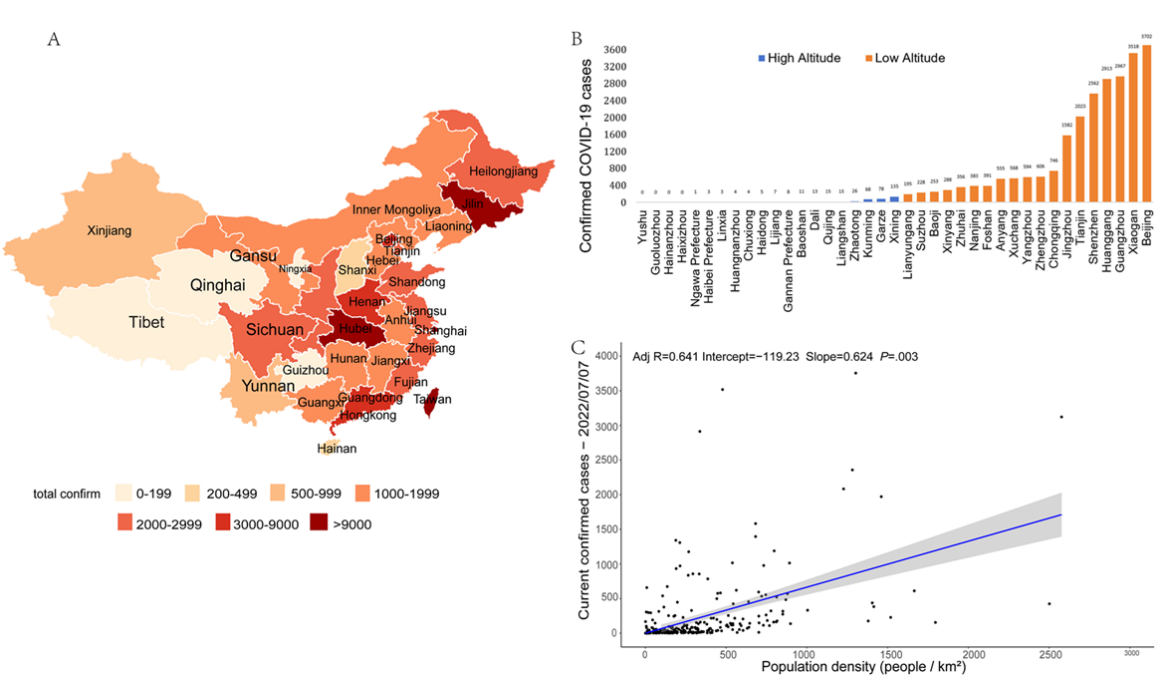
Pattern of confirmed COVID-19 cases at high altitudes within the study period. (A) Geographic patterns of confirmed COVID-19 cases in China as of July 7, 2022. (B) Comparison of confirmed COVID-19 cases reported in different cities at different altitudes. (C) Linear regression analysis between population density and confirmed COVID-19 cases in China.

During the study period from January 23, 2020, to June 7, 2022, a total of 732 COVID-19 cases were officially reported in the high-altitude region of China, which excluded one imported case in Tibet. The total numbers of confirmed COVID-19 cases were lower in the high-altitude regions, including Tibet, Qinghai, and Gansu, than in the low-altitude regions (Figure 1A); despite variation among regions, 98.4% (24,430/24,826) of the total COVID-19 cases were found in the lowland regions (Figure 1B). Taking into account the hidden factors of the dimension of population size (Multimedia Appendix 2) in different areas, a significant positive correlation between confirmed COVID-19 cases and population density (Figure 1C) was found during the study period.

### Correlation Between Meteorological Factors and Confirmed COVID-19 Cases in High- and Low-Altitude Regions

**Figure 2:**
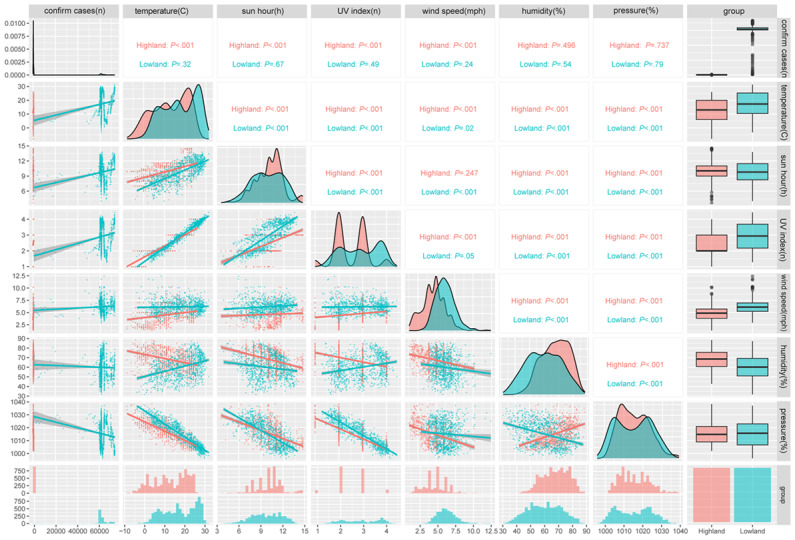
Spearman correlation analysis of confirmed COVID-19 cases and meteorological factors in high-altitude and low-altitude regions in China. The color represents each region group. Area charts represent frequencies and the scatterplots with lines represent the correlation. *P* values are based on 2-tailed tests; also see Multimedia Appendix 7.

To gain a better understanding of the correlation of meteorological factors with the spread of COVID-19 in high-altitude regions when compared with that in the lowlands, we performed a comparative correlation analysis of each variable (Figure 2); the detailed numeric results are provided in Multimedia Appendix 7. In the highlands of China, average temperature (*P=.*003), sunlight hours (*P*<.001), and UV index (*P*=.009) were negatively correlated with the number of confirmed COVID-19 cases, whereas the wind speed (ρ=0.388, *P*<.001) was positively correlated with confirmed cases.

### Correlation Between Air Quality Factors and Confirmed COVID-19 Cases in High- and Low-Altitude Regions

**Figure 3:**
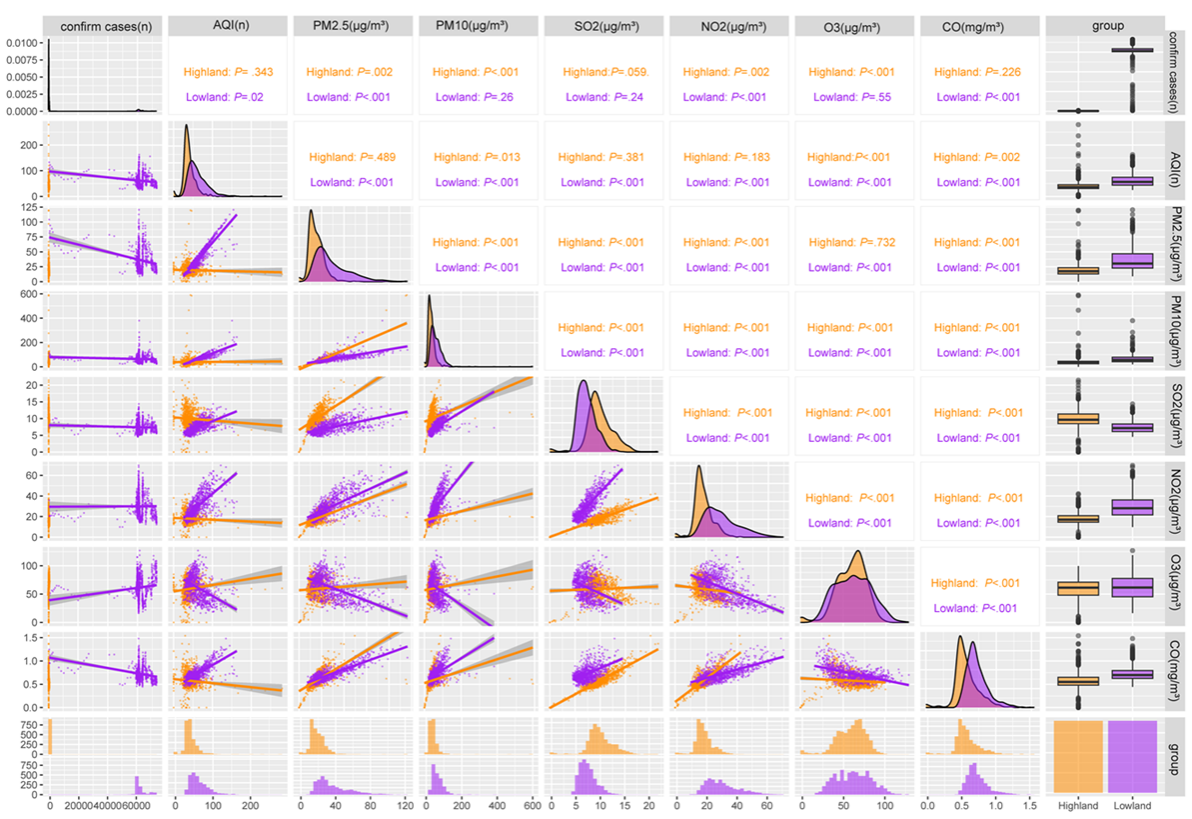
Spearman correlation analysis of confirmed COVID-19 cases and air quality factors in high- and low-altitude regions in China. The color represents each region group. Area charts represent frequencies and the scatterplot with lines represent correlations. AQI: air quality index; CO: carbon monoxide; NO2: nitrogen dioxide; O3: ozone; PM2.5: fine particulate matter; PM10: coarse particulate matter; SO2: sulfur dioxide. *P* values are based on 2-tailed tests; also see Multimedia Appendix 7.

As shown in Figure 3, we found significant correlations between air quality factors and COVID-19 cases at high and low altitudes. In the highlands, COVID-19 cases were positively correlated with fine particulate matter (PM2.5; *P*=.002), coarse particulate matter (PM10; *P<*.001), nitrogen dioxide (NO2; *P*=.02), and ozone (O3; *P*<.001). In the lowlands, a negative correlation was found between confirmed COVID-19 cases and air quality index (AQI; *P=*.01), whereas positive correlations were found for PM2.5 (*P*<.001), NO2 (*P*<.001), and carbon monoxide (CO; *P*<.001); in contrast to the pattern in the highlands, the average daily concentrations of PM10 and O3 were not significantly correlated with COVID-19 cases in lowland regions of China. The detailed results are shown in Multimedia Appendix 7.

### Correlations of Meteorological and Air Quality Factors With Normalized Confirmed COVID-19 Cases After Covariate Adjustment

The results of the mixed-effects multiple linear regression among the 12 provinces (Multimedia Appendix 2) at different altitudes are summarized in Table 1. In model 1, including each of the fixed-effect indicators in 12 separate models, a lower number of confirmed COVID-19 cases was significantly associated with average temperature, sunlight hours, UV index, air pressure, and air quality factors, including PM2.5, O3, and CO. However, in model 2, including all of the meteorological covariates considered simultaneously, only average temperature, UV index, and air pressure were significantly associated with the normalized confirmed COVID-19 cases. In model 3, including all of the air quality covariates considered simultaneously, only the average concentrations of O3 and CO were significantly correlated with confirmed COVID-19 cases. Finally, in model 4, including all 12 environmental indicators considered simultaneously, only average temperature, wind speed, and CO were significantly associated with confirmed COVID-19 cases.

**Table 1:** Mixed-effects multiple linear regression of the associations of normalized COVID-19 cases with meteorological and air quality factors including adjustment for covariates.

Fixed effects	Model 1^a^			Model 2^b^			Model 3^c^			Model 4^d^	
	Estimate (SE)	*P* value	Estimate (SE)		*P* value	Estimate (SE)		*P* value	Estimate (SE)		*P* value
Average temperature (°C)	–15.41 (2.55)	.001	–21.44 (7.47)		.004	—^e^		—	–27.90 (7.72)		.001
Sunlight hours	–45.31 (9.50)	.001	–12.55 (15.04)		.40	—		—	–8.86 (15.31)		.56
UV index	–201.24 (27.53)	.001	–257.41 (71.06)		.001	—		—	249.03 (71.84)		.05
Wind speed	7.934 (10.81)	.46	16.72 (11.27)		.13	—		—	16.71 (12.00)		.02
Humidity	–0.071 (1.42)	.96	–1.99 (1.81)		.27	—		—	1.9 8 (1.94)		.31
Pressure	17.64 (2.51)	.001	20.35 (5.51)		.001	—		—	24.32 (5.58)		.05
AQI^f^	–1.45 (0.67)	.03	—		—	–0.05 (0.88)		.94	0.11 (0.89)		.90
PM2.5^g^	4.71 (0.99)	.001	—		—	4.28 (1.35)		.15	2.96 (1.36)		.05
PM10^h^	0.36 (0.47)	.43	—		—	0.49 (0.58)		.39	0.52 (0.58)		.36
SO2^i^	6.93 (6.01)	.24	—		—	18.41 (6.75)		.64	22.06 (6.94)		.14
NO2^j^	1.88 (1.612)	.24	—		—	8.69 (1.97)		.14	10.21 (2.07)		.07
O3^k^	2.29 (0.77)	.002	—		—	2.09 (0.8)		.008	0.56 (0.86)		.51
CO^l^	375.4 (75.5)	.001	—		—	471.52 (101.55)		.001	564.31 (107.99)		.001

^a^Model 1 includes each of the fixed effects run in 12 separate models.

^b^Model 2 includes all meteorological covariates considered simultaneously.

^c^Model 3 includes all air quality covariates considered simultaneously.

^d^Model 4 includes all 12 environmental indicators considered simultaneously.

^e^Excluded from model.

^f^AQI: air quality index.

^g^PM2.5: fine particulate matter.

^h^PM10: coarse particulate matter.

^i^SO2: sulfur dioxide.

^j^NO2: nitrogen dioxide.

^k^O3: ozone.

^l^CO: carbon monoxide.

### Diversity of SARS-CoV-2 Sequences in High- and Low-Altitude Groups

**Figure 4:**
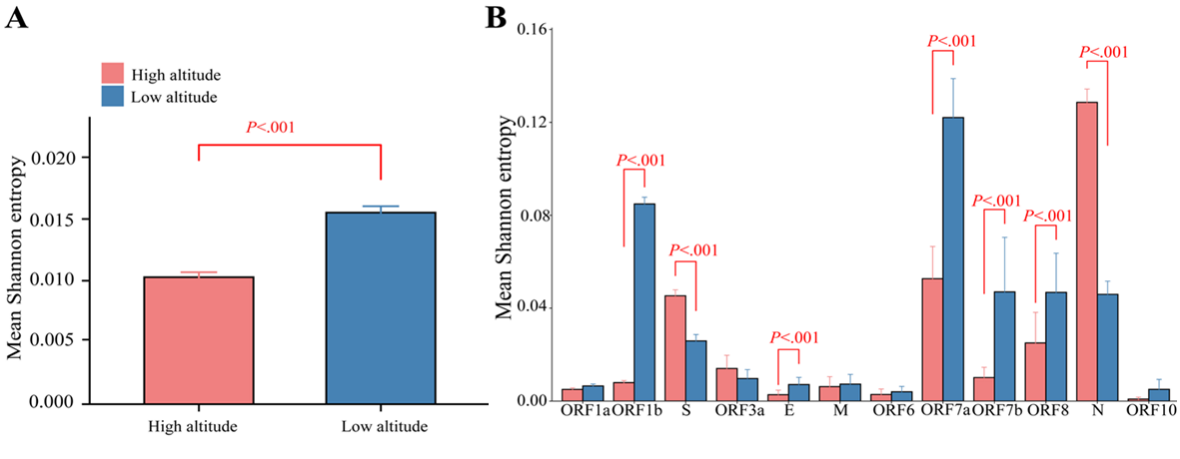
Mean sequence diversity across high-altitude (red) and low-altitude (blue) groups identified by Shannon entropy in nucleotides and different amino acids of the SARS-CoV-2 genome. (A) Mean sequence diversity identified by Shannon entropy in nucleotides of the whole SARS-CoV-2 genome. (B) Mean sequence diversity identified by Shannon entropy in different amino acids. Error bars indicate SEM. *P* values are based on the Wilcoxon signed rank test.

Comparable sequence diversity was found among the full-length SARS-CoV-2 genomes and 12 genes of SARS-CoV-2 in the high- and low-altitude groups (Figure 4). In the high-altitude group, the Sn values of nucleotides for each site were significantly lower than those in the low-altitude group (Figure 4A; *P*<.001). Likewise, the Sn values of amino acids for three open reading frames (ORFs; ORF1b, ORF7a, and ORF7b) in the high-altitude group were lower than those in the low-altitude group (Figure 4B; *P*<.001). Compared to those of the low-altitude group, the Sn values of the S and N genes were higher in the high-altitude group.

### Comparison of SARS-CoV-2 Gene Variant Frequencies in the High- and Low-Altitude Groups

**Figure 5:**
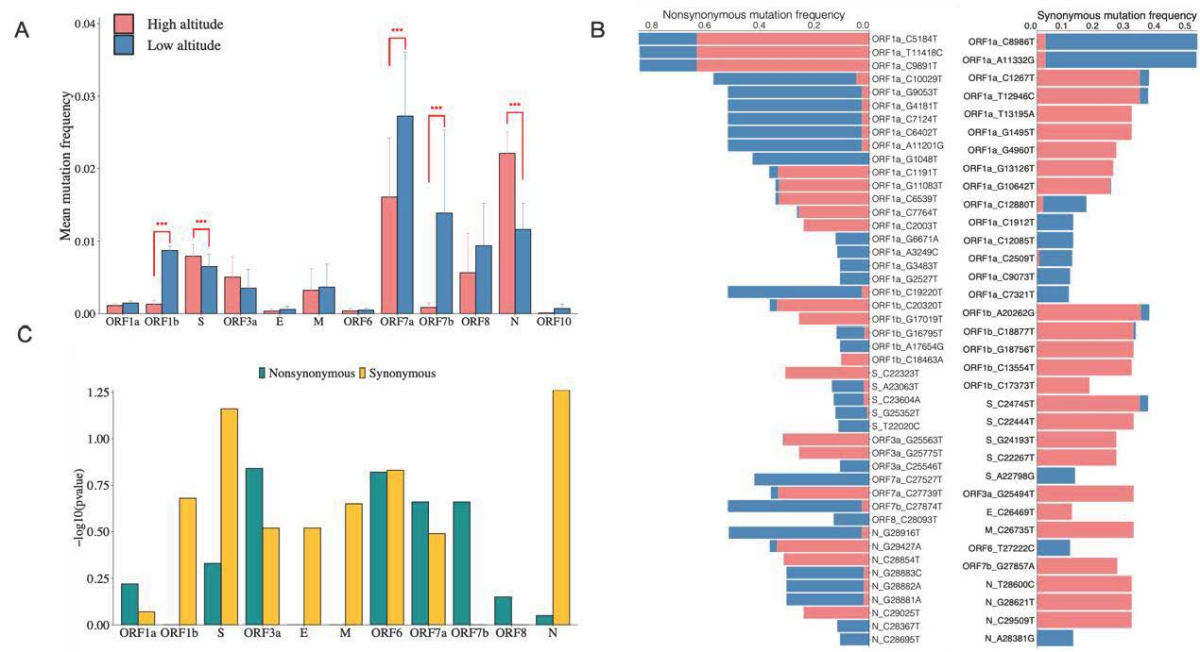
Distribution of mutations of SARS-CoV-2 in the high- and low-altitude groups. (A) Mean mutation frequency of open reading frames (ORFs) in the high- and low-altitude groups. (B) Comparison of frequencies of nonsynonymous and synonymous mutations between the high- and low-altitude groups. (C) Significance scores of nonsynonymous and synonymous mutations in each ORF. ***P<.001.

To determine the gene variant frequency difference between the high- and low-altitude groups, the Wilcoxon signed rank test was used to evaluate the significance of variant frequency in each of the ORFs (Figure 5A). The amino acid mutation frequencies for the three ORFs (ORF1b, ORF7a, and ORF7b) were significantly lower in the high-altitude group than in the low-altitude group (*P*<.001) but were higher for the S and N genes (*P*<.001). Among these, 44 nonsynonymous mutations and 32 synonymous mutations were found between the high- and low-altitude groups (Figure 5B; *P*<.001; mutation frequency>0.1). There was a greater proportion of nonsynonymous mutations in the low-altitude group than in the high-altitude group, whereas the opposite pattern was found for synonymous mutations. The –log10 *P* values for the differences in nonsynonymous and synonymous mutations for each ORF between the high- and low-altitude groups are shown in Figure 5C. Compared to the result in Figure 5A, the synonymous mutations of the S genes and N genes had relatively higher significance scores in the high-altitude group.

### Correlation Analysis Between Mutation Frequency and Environmental Factors

**Figure 6:**
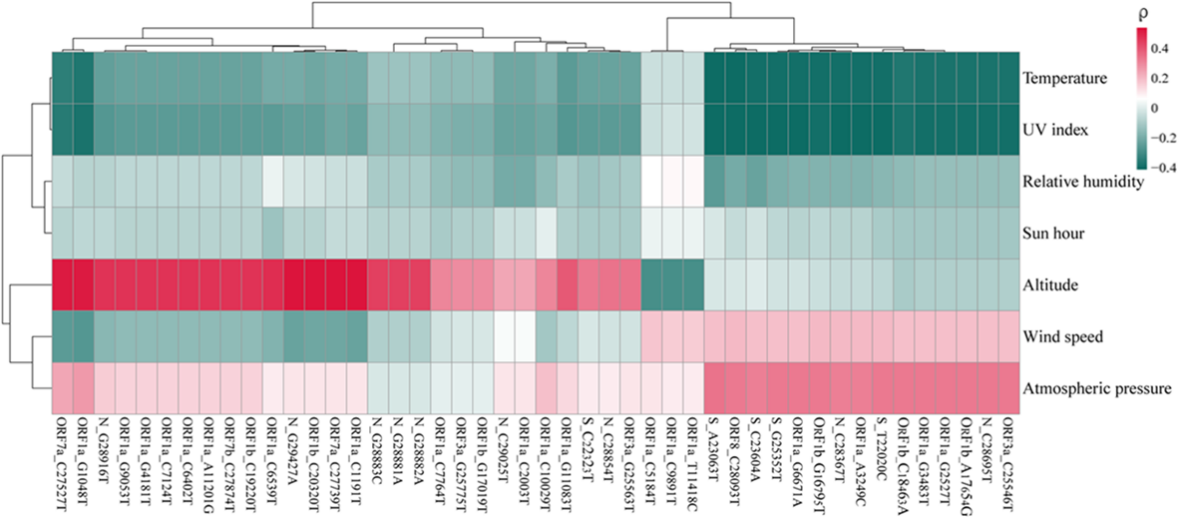
Spearman correlation coefficients of environmental factors and mutation frequencies among different sites in the high- and low-altitude groups. The color represents the strength of the Spearman correlation. Also see Multimedia Appendix 8.

The impact of 7 environmental factors on the nonsynonymous mutation frequency was analyzed for all sequences (Figure 6 and Multimedia Appendix 8). Most of the nonsynonymous mutations had a positive correlation with altitude, wind speed, and atmospheric pressure, but had negative correlations with UV index, relative humidity, and sunlight hours. The highest correlation between mutation frequency and environmental factors was found for altitude with N_G29427A, ORF1b_C20320T, and ORF1a_C1191T (ρ=0.53, *P*<.001); followed by UV index with ORF8_C28093T and S_A23063T (ρ=–0.42, *P*<.001); and temperature with ORF8_C28093T, S_C23604A, S_A23063T, and ORF1a_G6671A. Some significant nonsynonymous mutational events were also discovered in this study, including C22323T in the receptor-binding domain (RBD) of the S gene, which had a positive correlation with altitude (ρ=0.32, *P*<.001), indicating higher frequencies at high altitude. S gene mutations A23063T in the RBD region and C23604A in the fusion peptide (FP) region had higher frequencies at low altitude, and demonstrated a significant positive correlation with atmospheric pressure (ρ=0.34 and ρ=0.31, respectively; both *P*<.001) and a significant negative correlation with temperature and UV index (ρ=–0.42 and ρ=–0.41, respectively; both *P*<.001). Furthermore, co-occurring point mutations in the N gene, specifically G28881A, G28882A, and G28883C (R203K and G204R), had a higher mutation frequency at low altitude and were positively related to altitude (ρ=0.43, *P*<.001).

## Discussion

### Principal Findings

This study found that the lower number of confirmed COVID-19 cases in high-altitude regions of China may be related to population density and environmental factors. By further exploring SARS-CoV-2 sequences, we found different mutation frequencies in the high- and low-altitude regions, which were also correlated with environmental factors.

Population density is one of the most effective predictors related to the regional pandemic; a larger population greatly increases the infection and transmission rates of COVID-19 [28]. Therefore, our results suggest that when analyzing the correlation between COVID-19 cases in regions with different population dimensions and other possible factors, it is important to take into account the *normalized* number of COVID-19 cases to study the pandemic trend.

Meteorological factors may also influence COVID-19 transmission and cumulative infection in high-altitude regions. Previous studies indicated that higher temperature and UV radiation could contribute to a decrease in new cases of COVID-19 infection at high altitude [29-31]. This is supported by our Spearman correlation analysis showing that the average daily temperature, sunlight hours, and UV index were negatively related to the normalized COVID-19 cases in high-altitude regions. A high-altitude environment is distinguished by lower temperatures compared with those of low-altitude regions, along with significant differences between daylight hours, air dryness, and levels of UV light radiation [32-34]. Importantly, our study further found that the wind speed in the high-altitude region of China was significantly associated with the reducing trend of COVID-19 cases, which is consistent with earlier studies conducted in Italy, New York, and Singapore [2,35]. Analyzing the correlation between covariates in regions at different altitudes with different population dimensions showed positive correlations of average temperature and wind speed with normalized confirmed COVID-19 cases, in line with previous research.

Air pollution is widely recognized as a major risk factor for respiratory infection in humans, which has also played a significant role in the spread of COVID-19. Previous research suggested that the concentrations of NO2, PM2.5, PM10, and O3 are positively correlated with the number of confirmed COVID-19 cases [33,34,36-38]. Our Spearman correlation results align with these previous findings. NO2 is often linked with vehicle emissions and energy production [39], which is also an irritant of human respiratory diseases. Early research findings [40] support the results of this study, suggesting that travel restrictions should be among the specific actions implemented to reduce the spread of COVID-19. Previous studies indicated that PM2.5 and PM10 levels were positively correlated to the number of new daily confirmed COVID-19 cases in mainland China [41-43]. Consistently, our findings showed that lower average concentrations of PM2.5 and PM10 were related to a lower number of confirmed COVID-19 cases in the highlands.

It is clear that all of the above correlated factors may have influenced the cumulative infection trends of the pandemic in the highlands of China. The main sources of CO are motor vehicles and industrial source emissions; consequently, CO is closely related to population density and human activity. Therefore, taking into account the mixed correlations between all the covariates of focus in this study by including the related factors into a single mixed-effects model, among the air quality factors, only the average concentration of CO was positively associated with the normalized number of confirmed COVID-19 cases during the study period. This result also aligns with previous research conducted in China [43,44].

In terms of variations in the SARS-CoV-2 genome at high and low altitudes, the whole-genome sequences at high altitude showed lower diversity based on the mean Sn values of nucleotides and different amino acids compared to those in the low-altitude sites. Previous studies have suggested an impact of high altitude on the pathophysiology of COVID-19 [44,45], implying improved tolerance to SARS-CoV-2 infection for residents of high-altitude regions. Our study identified differences in the frequencies of SARS-CoV-2 sequence variants in various ORFs between high- and low-altitude regions, along with correlations between mutation frequency and environmental factors. Most of the nonsynonymous mutations identified have been reported previously and have important biological implications. We found that only the C22323T variant of the S gene had a positive correlation with altitude, with a greater frequency in the high-altitude group. Sivasubramanian et al [46] found that the S255F (C22323T) variant could reduce the affinity between the S protein and antibodies. We also identified the variant A23063T in the RBD region and the variant C23604A in the FP region of the S gene, with higher mutation frequencies detected at low altitude, showing a significantly positive correlation with atmospheric pressure and a strongly negative correlation with temperature and UV index. Previous studies found that the N501Y (A23063T) and Q493H variants enhanced the binding affinity to the human angiotensin converting-enzyme 2 receptor, thereby increasing infectivity [47], and that the Omicron peptide with variants N679K and P681H (C23604A) might increase viral infectivity and transmissibility [48]. These results suggest that atmospheric pressure, UV index, and temperature may affect the infectivity of COVID-19 driven by specific mutations of the S gene in low-altitude regions. In addition, three co-occurring variants (G28881A, G28882A, G28883C) in the N gene had higher mutation frequencies in the low-altitude group and were positively related to altitude; these mutations were previously reported to destabilize and decrease the overall structural flexibility of the SARS-CoV-2 genome [48].

Notably, the potential impact of the strategy implemented by the government in response to the pandemic in high-altitude regions on the observed differences should not be ignored. This study only included data collected from January 22, 2020, to March 19, 2021, during which time there was only one imported COVID-19 case in Tibet that was excluded from the analysis. However, after several consecutive months of no new cases, a newly confirmed case was reported in Tibet on August 7, 2022. Since then, eight new cases have been added with a total of 1437 confirmed cases reported to date [21]. Several reasons for this new outbreak are worth discussing. First, the virus underlying the epidemic in Tibet is Omicron BA.2.76, which has characteristics of strong, fast transmission ability and the potential to more readily escape from immune protection. Second, the first discovered infection on August 7 was a close contact of a family, with suspected transmission occurring at a family gathering, and August is the peak tourist season in Tibet. Thus, under the conditions of relatively relaxed travel restrictions, the close contact and population flow greatly increased the spread of the virus. Third, the lack of epidemic prevention and medical appliances in high-altitude regions limited access to health care systems, and the limited capacity for viral testing and contact tracing worsened the situation of the pandemic in Tibet. Therefore, effective and accurate government policies have played an important role in preventing Tibet from being affected by the epidemic, and the government policy in response to the pandemic must be considered; indeed, the response needs to be strengthened, although China indicated that they had ceased counting COVID-19 cases on December 23, 2022, and stated that the COVID-19 pandemic is effectively now over [49].

### Limitations

Our study has some limitations. First, among the data available for confirmed COVID-19 cases, we only excluded the one known imported case in Tibet during the study period as of March 19, 2021, whereas the collected COVID-19 data from the other four areas may include both local and imported cases. This is mainly due to a lack of accurate publicly available COVID-19 data at the city level. Similarly, the incompleteness of some city-level information of genome sequences also led to difficulties in studying the differences in genetic variants among cities in the same province. Second, we aimed to evaluate whether virus mutations are associated with particular viral lineages. The sequences of SARS-CoV-2 in the high- and low-altitude groups represented 33 different virus lineages (Multimedia Appendix 9); B.1.617.2, AY.122, and B.1.36.16 were the major lineages, and the mutations of each lineage in which the mutation frequency was greater than 0.5 are listed in Multimedia Appendix 10. The number of different lineages between high- and low-altitude groups is also unbalanced. Third, we lacked the clinical information to study the potential relationships between mutations and clinical outcomes of infection at high and low altitudes. Therefore, future studies should focus on exploring the underlying mechanisms contributing to the links between patterns of SARS-CoV-2 mutation and case numbers at different altitudes.

### Conclusions

Compared to that in the lowland area of China, the total number of confirmed COVID-19 cases in the highland was substantially lower. Population density and environmental factors, including average temperature, sunlight hours, UV index, wind speed, NO2, O3, PM2.5, and CO, were identified as indicators with a significant influence on the cumulative pandemic trend in the highlands. Among these factors, average temperature and CO were identified as the major meteorological and air quality factors associated with the spread of COVID-19 infection in China. Furthermore, we identified different mutations from SARS-CoV-2 isolates between high- and low-altitude regions, and there was a significant impact of environmental factors on virus mutation. Overall, this study adds important knowledge of the impact of altitude and related environmental factors on the cumulative infection rate of COVID-19, providing novel suggestions for preventive interventions.

## Appendix

Multimedia Appendix 1 Key environmental factors associated with respiratory infectious diseases reported in the literature.

Multimedia Appendix 2 Detailed characteristics of the regions included in the study area in the high- and low-altitude groups.

Multimedia Appendix 3 Descriptive statistics of all variables collected during the study period.

Multimedia Appendix 4 Description of all variables analyzed in this study.

Multimedia Appendix 5 SARS-CoV-2 sequences downloaded from the GISAID database for analysis in this study.

Multimedia Appendix 6 One-sample Kolmogorov-Smirnov test of normality for each variable.

Multimedia Appendix 7 Spearman correlation analysis of meteorological factors and air quality factors with confirmed COVID-19 cases in high-altitude and low-altitude regions.

Multimedia Appendix 8 Spearman correlation analysis of environmental factors and mutation frequency in the high- and low-altitude groups.

Multimedia Appendix 9 Frequencies of nonsynonymous and synonymous mutations of the main lineages of SARS-CoV-2 in the high- and low-altitude groups.

Multimedia Appendix 10 Number of samples of different SARS-CoV-2 lineages in the high-altitude (red) and low-altitude (blue) regions.

## Abbreviations

AQI: air quality index
CO: carbon monoxide
FP: fusion peptide
GISAID: Global Initiative on Sharing All Influenza Data
NO2: nitrogen dioxide
O3: ozone
ORF: open reading frame
PM2.5: fine particulate matter
PM10: coarse particulate matter
RBD: receptor-binding domain
Sn: Shannon entropy

## Data Availability

The data sets generated and analyzed during the study are provided in the Multimedia Appendix files.
