# Correction: Influence of Environmental Factors and Genome Diversity on Cumulative COVID-19 Cases in the Highland Region of China: Comparative Correlational Study

**DOI:** 10.2196/59070

**Published:** 2024-04-10

**Authors:** Zhuoga Deji, Yuantao Tong, Honglian Huang, Zeyu Zhang, Meng Fang, M James C Crabbe, Xiaoyan Zhang, Ying Wang

**Affiliations:** 1 Research Center for Translational Medicine, Shanghai East Hospital, School of Life Sciences and Technology, Tongji University Shanghai China; 2 Information School, The University of Sheffield Sheffield United Kingdom; 3 Department of Clinical Laboratory Medicine Center Yueyang Hospital of Integrated Traditional Chinese and Western Medicine, Shanghai University of Traditional Chinese Medicine Shanghai China; 4 Department of Laboratory Medicine, Shanghai Eastern Hepatobiliary Surgery Hospital Shanghai China; 5 Wolfson College, Oxford University Oxford United Kingdom; 6 Institute of Biomedical and Environmental Science & Technology, University of Bedfordshire Bedfordshire United Kingdom; 7 School of Life Sciences, Shanxi University Shanxi China

In “Influence of Environmental Factors and Genome Diversity on Cumulative COVID-19 Cases in the Highland Region of China: Comparative Correlational Study” (Interact J Med Res 2024;13:e43585) the authors noted one error.

In the originally published manuscript, author Zhuoga Deji was noted as having contributed to the manuscript as the first author.

This has been corrected to note that both authors Zhuoga Deji and Yuantao Tong contributed equally to the manuscript, and they should be regarded as joint first authors.

The correction will appear in the online version of the paper on the JMIR Publications Interactive Journal of Medical Research publication website on April 10, 2024, together with the publication of this correction notice. Because this was made after submission to PubMed, PubMed Central, and other full-text repositories, the corrected article has also been resubmitted to those repositories.

