# Supplementary material for: Influence of Environmental Factors and Genome Diversity on Cumulative COVID-19 Cases in the Highland Region of China: Comparative Correlational Study
Source: Interact J Med Res. 2024 Mar 25;13:e43585. doi: 10.2196/43585 (PMC10964983; doi:10.2196/43585)
Supplement: Multimedia Appendix 1 [file ijmr_v13i1e43585_app1.docx]

**Multimedia Appendix 1. Environmental factors obtained from the respiratory infectious diseases literature.**
