# Supplementary material for: Influence of Environmental Factors and Genome Diversity on Cumulative COVID-19 Cases in the Highland Region of China: Comparative Correlational Study
Source: Interact J Med Res. 2024 Mar 25;13:e43585. doi: 10.2196/43585 (PMC10964983; doi:10.2196/43585)
Supplement: Multimedia Appendix 2 [file ijmr_v13i1e43585_app2.docx]

| **Multimedia Appendix 2. Description of *GP*: group; *PV*:province; *CT*:city; *AT*: altitude(average m); *P*: population (thousand persons); *LA*: land area(km^2^), respectively for the study area.** | | | | | |
| --- | --- | --- | --- | --- | --- |
| *GP* | *PV* | *CT* | *AT* | *P* | *LA* |
| High  Altitude | Qinghai | Haibei Prefecture | 2833 | 265.3 | 3443 |
|  |  | Xining | 2676 | 2468 | 7524 |
|  |  | Yushu | 4403 | 425.2 | 204891 |
|  |  | Guoluozhou | 4300 | 215.6 | 74367 |
|  |  | Haidong | 2114 | 1358.5 | 13202 |
|  |  | Hainanzhou | 3000 | 447 | 43493 |
|  |  | Haixizhou | 3200 | 468.2 | 300854 |
|  |  | Huangnanzhou | 2480 | 276.2 | 17809 |
|  | Yunnan | Baoshan | 1760 | 2431 | 19637 |
|  |  | Chuxiong | 1879 | 2417 | 29300 |
|  |  | Dali | 2175 | 3338 | 29500 |
|  |  | Kunming | 1964 | 8460 | 21000 |
|  |  | Lijiang | 2673 | 1254 | 20600 |
|  |  | Qujing | 1646 | 5766 | 29000 |
|  |  | Zhaotong | 1705 | 5093 | 23000 |
|  | Sichuan | Ngawa Prefecture | 3143 | 822.6 | 85131 |
|  |  | Garzê | 3165 | 1107 | 152629 |
|  |  | Liangshan | 2437 | 4858 | 60423 |
|  | Gansu | Gannan Prefecture | 3033 | 691.8 | 38521 |
|  |  | Linxia | 2636 | 2109.8 | 8169 |
| Low Altitude | Guangdong | Foshan | 8 | 9500 | 3798 |
|  |  | Guangzhou | 21 | 18680 | 7249 |
|  |  | Shenzhen | 48 | 17560 | 1998 |
|  |  | Zhuhai | 20 | 2440 | 1737 |
|  | Henan | Xinyang | 143 | 6234 | 18916 |
|  |  | Anyang | 102 | 5478 | 7352 |
|  |  | Xuchang | 78 | 4380 | 4979 |
|  |  | Zhengzhou | 162 | 12600 | 7568 |
|  | Jiangsu | Nanjing | 25 | 9315 | 6586 |
|  |  | Yangzhou | 10 | 4560 | 6504 |
|  |  | Suzhou | 6 | 12750 | 8395 |
|  |  | Lianyungang | 18 | 4600 | 7614 |
|  | Hubei | Wuhan | 48 | 42327 | 8483 |
|  |  | Xiaogan | 30 | 4270 | 8941 |
|  |  | Jingzhou | 156 | 1079 | 1582 |
|  |  | Huanggang | 143 | 5883 | 17453 |
|  | Shanxi | Baoji | 94 | 3321.9 | 18120 |
|  | Beijing | Beijing | 47 | 21890 | 16807 |
|  | Tianjin | Tianjin | 28 | 13866 | 11300 |
|  | Chongqing | Chongqing | 754 | 32050 | 83000 |
