# Supplementary material for: Influence of Environmental Factors and Genome Diversity on Cumulative COVID-19 Cases in the Highland Region of China: Comparative Correlational Study
Source: Interact J Med Res. 2024 Mar 25;13:e43585. doi: 10.2196/43585 (PMC10964983; doi:10.2196/43585)
Supplement: Multimedia Appendix 3 [file ijmr_v13i1e43585_app3.docx]

| **Multimedia Appendix 3. Descriptive statistic of all of variables during study period** | | | | |
| --- | --- | --- | --- | --- |
| N(9867) | Minimum | Maximum | Mean | Std. Deviation |
| altitude | 14.75 | 3126.00 | 1024.59 | 1303.19 |
| Population_density | 8.90 | 3259.00 | 860.51 | 928.25 |
| normalized_cases | 0.00 | 38.70 | 4.49 | 10.74 |
| Total_confirm | 0.00 | 56855 | 5564.72 | 15962.48 |
| avgtempC | -19.00 | 34.00 | 14.95 | 9.80 |
| sunHour | 3.50 | 14.50 | 9.78 | 2.43 |
| uvIndex | 1.00 | 5.00 | 2.73 | 0.87 |
| windspeedMiles | 1.00 | 20.00 | 6.06 | 2.38 |
| humidity | 10.12 | 99.00 | 61.39 | 18.74 |
| pressure | 991.50 | 1044.00 | 1014.95 | 9.03 |
| AQI | 1.00 | 445.35 | 48.53 | 35.88 |
| PM2.5 | 1.00 | 237.75 | 27.43 | 24.43 |
| PM10 | 1.00 | 1564.710 | 49.24 | 50.16 |
| SO2 | 1.0 | 43.79 | 7.78 | 4.26 |
| NO2 | 1.00 | 99.80 | 23.42 | 14.94 |
| O3 | 1.00 | 178.21 | 54.11 | 29.62 |
| CO | 0.10 | 2.96 | 0.69 | 0.32 |
