# Supplementary material for: Influence of Environmental Factors and Genome Diversity on Cumulative COVID-19 Cases in the Highland Region of China: Comparative Correlational Study
Source: Interact J Med Res. 2024 Mar 25;13:e43585. doi: 10.2196/43585 (PMC10964983; doi:10.2196/43585)
Supplement: Multimedia Appendix 4 [file ijmr_v13i1e43585_app4.docx]

| **Multimedia Appendix 4. The description of all of variables used in this research** | | | |
| --- | --- | --- | --- |
| **Name** | **Nature** | **Description** | Factors |
| Province | String | Name of province |  |
| Date | Date | Observation date |  |
| altitude | String | Average altitude for each study area (m) |  |
| Population_density | numeric | Population size/land area (km2) |  |
| **Dependent variable** | | | |
| normalized_cases | numeric | Total cases / population density |  |
| Total_confirm | numeric | Total confirmed cases |  |
| **Independent variable** | | | |
| avgtempC | numeric | Daily average temperature (0C) | Meteorological Factors |
| sunHour | numeric | Daily average Sunlight hour |  |
| uvIndex | numeric | Daily average UV index |  |
| windspeedMiles | numeric | Daily average wind speed |  |
| humidity | numeric | Daily average relative humidity |  |
| pressure | numeric | Daily average air pressure |  |
| AQI | numeric | Daily average air quality index | Air quality factor |
| PM2.5 | numeric | Daily average particulate mater (2.5) |  |
| PM10 | numeric | Daily average particulate mater (10) |  |
| SO2 | numeric | Daily average aerosol optical thickness |  |
| NO2 | numeric | Daily average nitrogen dioxide |  |
| O3 | numeric | Daily average ozone |  |
| CO | numeric | Daily average carbon monoxide |  |
