# Supplementary material for: Influence of Environmental Factors and Genome Diversity on Cumulative COVID-19 Cases in the Highland Region of China: Comparative Correlational Study
Source: Interact J Med Res. 2024 Mar 25;13:e43585. doi: 10.2196/43585 (PMC10964983; doi:10.2196/43585)
Supplement: Multimedia Appendix 10 [file ijmr_v13i1e43585_app10.docx]

**The number of samples in different SARS-CoV-2 lineages. The red represents high altitude, the blue represents low altitude.**
